# Association between lifetime coffee consumption and late life cerebral white matter hyperintensities in cognitively normal elderly individuals

**DOI:** 10.1038/s41598-019-57381-z

**Published:** 2020-01-16

**Authors:** Jeongbin Park, Ji Won Han, Ju Ri Lee, Seonjeong Byun, Seung Wan Suh, Jae Hyoung Kim, Ki Woong Kim

**Affiliations:** 1 Department of Brain and Cognitive Science, Seoul National University College of Natural Sciences, Seoul, Korea; 2 Department of Neuropsychiatry, Seoul National University Bundang Hospital, Seongnam, Korea; 3 Department of Radiology, Seoul National University Bundang Hospital, Seongnam, Korea; 4 Department of Radiology, Seoul National University College of Medicine, Seoul, Korea; 5 Department of Psychiatry, Seoul National University College of Medicine, Seoul, Korea

**Keywords:** Cognitive ageing, Neurodegeneration, White matter disease

## Abstract

Coffee consumption is associated with cerebral hypoperfusion that may contribute to the development of cerebral white matter hyperintensities (WMH). We investigated the effect of lifetime coffee consumption on the volume of WMH (V_WMH_) in late life, and compared the effect between men and women since caffeine clearance may be different between sexes. We enrolled 492 community-dwelling cognitively normal elderly individuals (73.4 ± 6.7 years old on average) from the Korean Longitudinal Study on Cognitive Aging and Dementia. We evaluated their patterns and amounts of coffee consumption using a study-specific standardized interview and estimated cerebral V_WMH_ by automatic segmentation of brain fluid-attenuated inversion recovery sequence magnetic resonance images. Higher cumulative lifetime coffee consumption was associated with higher logV_WMH_ in both sexes (p = 0.030). The participants who consumed more than 2 cups of coffee per day on average in their lifetime showed higher logV_WMH_ in late life than those who consumed less. When both sexes were analyzed separately, these coffee-logV_WMH_ associations were found only in women, although the volumes of brain and white matter of women were smaller than those of men. Our findings suggest that prolonged high coffee consumption may be associated with the risk of WMH in late life.

## Introduction

Although numerous studies have linked coffee consumption with lower risk of stroke, diabetes mellitus (DM), cardiovascular disease (CVD), and reduced risk of developing cognitive impairment and/or dementia^1–5^, health impacts of coffee consumption on human brain has remained controversial. Coffee is a major dietary source of caffeine around the world^6^, and caffeine (1, 3, 7-trimethylxanthine) is associated with cerebral hypoperfusion in humans^7^. Regular caffeine consumption, of 2 to 4 cups of coffee a day, eliminates the protective effect of ischemic preconditioning^8^ and reduces global cerebral blood flow by approximately 22–30%^9–12^. Long-term caffeinated coffee increases blood pressure^6,13,14^, increases vascular resistance^15^, and causes arterial stiffness^16^ and cerebral vasoconstriction^12^.

White matter hyperintensities (WMH) are brain areas in the white matter (WM) that appear abnormally hyperintense on T2-weighted or fluid-attenuated inversion recovery (FLAIR) sequences of brain magnetic resonance imaging (MRI)^17^. Although the etiologies of WMH are not fully understood, increasing evidence suggests that a majority of WMH may be attributable to cerebral hypoperfusion and ischemic brain damages^17–21^. Hyperintense WM showed lower cerebral blood flow and cerebrovascular reactivity than normal-appearing WM^22^, and were associated with high blood pressure, DM, other CVD, stroke and cognitive impairments^17–21^.

Therefore prolonged coffee consumption may increase the risk of WMH in late life. Furthermore, the effects of coffee on the risk of WMH may be more pronounced in women than in men, since estradiol decreases caffeine clearance in humans^23^. Temple and Ziegler reported that women show greater blood pressure responses to caffeine than do men, possibly due to differences in the levels of circulating sex hormones^24^. Elderly women have greater WMH volume relative to their WM volume compared to men^25^, and have a greater progression of WMH than men^26^. This study aimed to investigate the association between the amount of lifetime coffee consumption and the volume of WMH (V_WMH_) in cognitively normal elderly individuals, and to examine whether this association is different between sexes.

## Methods

### Study participants

In this retrospective cohort study, we enrolled 492 community-dwelling cognitively normal elderly Koreans (204 men and 288 women) aged 60 years or above (73.4 ± 6.7 years old) at Seoul National University Bundang Hospital for the Korean Longitudinal Study on Cognitive Aging and Dementia (KLOSCAD)^27^. The KLOSCAD is a nationwide population-based prospective elderly cohort study that was launched in 2009. In the KLOSCAD, a total of 6,818 community-dwelling Koreans aged 60 years or older were randomly sampled from 30 villages and towns across South Korea using residential rosters. The baseline evaluation was conducted from November 2010 to October 2012, and follow-up evaluations were conducted every two years^27^.

All participants were fully informed regarding study participation, and written informed consent was obtained from the participants or their legal guardians. The Institutional Ethics Review Board of the Seoul National University Bundang Hospital approved the study protocol.

### Assessments of clinical characteristics and diagnosis

Geriatric psychiatrists with expertise in dementia research administered in person standardized diagnostic interviews including detailed medical histories, physical and neurological examinations, laboratory tests, echocardiography and chest X-ray according to the Korean version of the Consortium to Establish a Registry for Alzheimer’s Disease Assessment Packet (CERAD-K) Clinical Assessment Battery (CERAD-K-C)^28^ and the Korean version of the Mini International Neuropsychiatric Interview^29^. A research neuropsychologist or trained research nurse administered the CERAD-K Neuropsychological Assessment Battery (CERAD-K-N)^28,30^, Digit Span Test^31^, and Frontal Assessment Battery^32^ to each participant. Trained research nurses collected data on body mass index (BMI), history of hypertension, diabetes mellitus (DM), and cardiovascular disease (CVD), amount of lifetime alcohol consumption (ALAC, standard unit-years), amount of lifetime smoking (ALS, pack-years), Geriatric Depression Scale (GDS)^33^, Pittsburgh Sleep Quality Index (PSQI)^34^, Cumulative Illness Rating Scale (CIRS)^35^, and obstructive sleep apnea as a STOP questionnaire^36^. We diagnosed dementia and other Axis I mental disorders. We defined normal cognition as 0 in Clinical Dementia Rating^37^ without any of following conditions; dementia and major psychiatric disorders according to the Diagnostic and Statistical Manual of Mental Disorders, 4^th^ Edition, Text Revision (DSM-IV-TR) criteria^38^, mild cognitive impairment according to the consensus criteria from the International Working Group^39^, a history of stroke or transient ischemic attack, neurologic diseases, substance abuse or dependence, and any history of brain tumors.

### Assessment of coffee consumption

We evaluated the pattern and amount of coffee consumption using a study-specific interview that examined the average amount of daily coffee consumption over the past year (current ADCC, cups/d), the average amount of daily coffee consumption during their lifetime (ADCC, cups/d), and their ages at the start and end of coffee drinking (years). We calculated the duration of lifetime coffee consumption (DLCC, years) by subtracting the age at which coffee drinking started from the age at which coffee drinking ended. We estimated the amount of lifetime coffee consumption (ALCC, cup-years) by multiplying the ADCC by the DLCC (see Supplementary Table). Based on the previous literatures showing the effects of 200–250 mg caffeine (equivalent to 2 cups of coffee) on the cerebrovascular health^7–12,14^, we classified the participants into three groups according to their ADCC: never consumed (n = 101, 20.5%), consumed two cups per day or less (n = 306, 62.2%), and consumed over 2 cups per day (n = 85, 17.3%). We assumed that the participants did not change their coffee consumption habits during their lifetime.

### Assessment of V_WMH_

We performed brain MRI using a 3.0 Tesla GE SIGNA Scanner (GE Healthcare; Milwaukee, WI) within three months of the clinical assessments. We obtained three-dimensional structural T1 weighted spoiled gradient echo sequences (acquisition voxel size = 1.0 × 0.5 × 0.5 mm; 1.0-mm sagittal slices thickness with no inter-slice gap; repetition time = 25.0 ms; echo time = 3.68 ms; number of excitations = 1; flip angle = 90°; field of view = 240 × 240 mm; and acquisition matrix size = 175 × 256 × 256 mm) and FLAIR sequences (acquisition voxel size = 0.5 × 0.5 × 3.0 mm; 3.0-mm axial slices thickness with no inter-slice gap; repetition time = 9,900 ms; echo time = 160 ms; inversion time = 2,500 ms; number of excitations = 1; flip angle = 90°; field of view = 240 × 240 mm; and acquisition axial plane matrix size = 256 × 256 mm). We quantified the absolute whole brain V_WMH_ using a fully automated monospectral and intensity-based segmentation method from the FLAIR sequence, as described in a previous report^40^. We obtained the total intracranial volume (ICV) by summing the volumes of total grey matter, WM, and cerebrospinal fluid using the Freesurfer software (version 5.3.0; http://surfer.nmr.mgh.harvard.eu). We implemented all study procedures using custom written codes running in MATLAB R2014a (The Math Works, Inc., Natick, MA, USA) as well as functions from the Statistical Parametric Mapping software (version 8, SPM8; Wellcome Trust Centre for Neuroimaging, London; http://www.fil.ion.ucl.ac.uk/spm). All analyses were performed blind to participant details, including demographics, clinical characteristics, and coffee consumption data.

### Statistical analysis

We compared continuous variables using one-way analyses of variance and categorical variables using chi-square tests between groups. Given the skewed distribution of the V_WMH_, we normalized the V_WMH_ by dividing the corresponding ICV and log-transformed the values for better approximation of normality (logV_WMH_). In each group (all, men, and women), we performed two models (ALCC with covariates in the MODEL A, and ADCC and DLCC with covariates in the MODEL B) because ALCC is the value multiplied by ADCC and DLCC. We examined the effect of ALCC on logV_WMH_ using multiple linear regression models adjusted for the following potential confounding factors: age, sex, years of education, BMI, hypertension, DM, CVD, ALAC, ALS, GDS score, PSQI score, and CIRS score (MODEL A). In examining the effects of ADCC and DLCC on logV_WMH_, we also conducted multiple linear regression analysis that computed ADCC and DLCC as independent variables, to determine which variable more strongly predicted logV_WMH_ (MODEL B). In these analyses, we adjusted for the covariates of age, sex, years of education, BMI, hypertension, DM, CVD, ALAC, ALS, GDS score, PSQI score, and CIRS score. In the regression analyses, the ALCC, ADCC, and DLCC values were entered as continuous variables. We assessed multicollinearity using collinearity statistical tests (tolerance and variance inflation factor). We then compared logV_WMH_ among the three groups of ADCC (never, ≤2.0 cups/d, and >2.0 cups/d) using analyses of covariance with Bonferroni post hoc comparisons. In these analyses, we adjusted for the covariates of age, sex, years of education, BMI, hypertension, DM, CVD, ALAC, ALS, GDS score, PSQI score, CIRS score, and DLCC. For all analyses, a two-sided p value less than 0.05 was considered to be statistically significant, and Bonferroni corrections were employed in multiple comparisons. We performed all statistical analyses using the Statistical Package for the Social Sciences (SPSS) for Windows (version 20.0; IBM Corporation; Armonk, NY).

## Results

**Figure 1:**
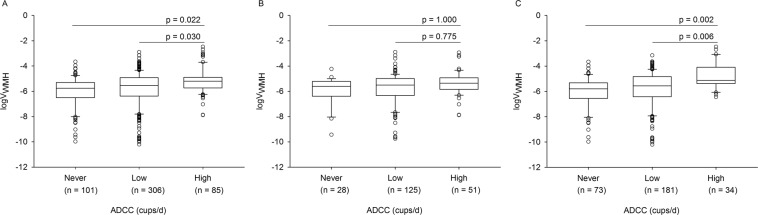
Comparisons of logV_WMH_ between the participants grouped by ADCC for (**A**) All (n = 492), (**B**) men (n = 204), and (**C**) women (n = 288)^*^. ^*^Analyses of covariance adjusted for age, years of education, body mass index (BMI), hypertension, diabetes mellitus (DM), cardiovascular disease (CVD), amount of lifetime alcohol consumption (ALAC), amount of lifetime smoking (ALS), Geriatric Depression Scale (GDS), Pittsburgh Sleep Quality Index (PSQI), Cumulative Illness Rating Scale (CIRS) and duration of lifetime coffee consumption (DLCC). Notes: logV_WMH_ = log-transformed white matter hyperintensities volume/intracranial volume; ADCC = average amount of daily coffee consumption during lifetime; Low = 2 cups of coffee or less per day; High = more than 2 cups of coffee per day.

The participant characteristics are summarized in Table 1. ALCC was associated with logV_WMH_ (adjusted R^2^ = 0.181, standardized β = 0.094, p = 0.030; Table 2; MODEL A). V_WMH_ increased by 0.044 cm^3^ as the coffee consumption increased by one cup-year. The association between logV_WMH_ and ADCC was significant (adjusted R^2^ = 0.186, standardized β = 0.126, p = 0.008) while the association between logV_WMH_ and DLCC was not statistically significant (adjusted R^2^ = 0.201, standardized β = −0.003, p = 0.958 for DLCC; Table 2; MODEL B). V_WMH_ increased by 1.251 cm^3^ as the average daily coffee consumption increased by one cup. When we divided the participants into three groups according to ADCC, the participants who consumed an average of more than 2 cups of coffee per day had a greater logV_WMH_ than those who consumed an average of 2 cups of coffee per day or less as well as those who never consumed coffee (Fig. 1).

**Table 1:** Participant characteristics.

	All	Never	Low	High	Statistics	
	(n = 492)	(n = 101)	(n = 306)	(n = 85)	p^a^	Post hoc^b^
Women, n (%)	288 (58.5)	73 (72.3)	181 (59.2)	34 (40.0)	<0.001	—
Age (years, mean ± SD)	73.4 (6.7)	73.6 (6.1)	73.2 (7.1)	73.6 (6.4)	0.849	—
Education (years, mean ± SD)	10.9 (5.3)	9.6 (5.2)	11.0 (5.2)	11.8 (5.4)	0.009	—
Body Mass Index (kg/m^2^, mean ± SD)	23.8 (3.0)	22.6 (2.6)	24.1 (3.2)	24.0 (2.7)	<0.001	1 < 02, 3
Total intracranial volume (cm^3^, mean ± SD)	1558.6 (155.3)	1508.9 (148.9)	1562.6 (153.1)	1602.9 (156.6)	<0.001	1 < 02, 3
Hypertension, n (%)	259 (52.6)	58 (57.4)	156 (51.0)	45 (52.9)	0.530	—
Diabetes mellitus, n (%)	107 (21.7)	23 (22.8)	68 (22.2)	16 (18.8)	0.767	—
Cardiovascular disease, n (%)	62 (12.6)	11 (10.9)	43 (14.1)	8 (9.4)	0.441	—
ALAC (standard unit-years, mean ± SD)	24.9 (61.6)	18.4 (55.0)	24.1 (58.2)	35.1 (78.1)	0.173	—
ALS (pack-years, mean ± SD)	9.0 (17.6)	5.4 (17.0)	7.7 (15.3)	17.9 (22.9)	<0.001	1, 2 < 3
Geriatric Depression Scale (points, mean ± SD)	10.6 (6.7)	11.9 (7.2)	10.3 (6.7)	9.7 (6.2)	0.049	—
Pittsburgh Sleep Quality Index (points, mean ± SD)	6.9 (3.6)	7.3 (3.5)	6.7 (3.5)	7.1 (3.7)	0.236	—
STOP questionnaire (points, mean ± SD)	1.1 (0.9)	1.2 (0.9)	1.1 (0.9)	1.2 (0.9)	0.343	—
Cumulative Illness Rating Scale (points, mean ± SD)	6.0 (3.1)	6.0 (2.78)	6.0 (2.9)	6.3 (3.7)	0.658	—
Age at onset of coffee consumption (years, mean ± SD)	38.4 (16.9)	—	39.6 (17.6)	33.8 (13.4)	—	—
Age at end of coffee consumption (years, mean ± SD)	68.1 (10.6)	—	68.1 (9.3)	68.0 (13.7)	—	—
DLCC (years, mean ± SD)	26.7 (20.1)	0.0 (0.0)	32.7 (17.0)	36.9 (14.8)	<0.001	1 < 2, 3
Current ADCC (cups/d, mean ± SD)	1.3 (1.2)	0.0 (0.0)	1.3 (0.9)	2.6 (1.3)	<0.001	1 < 2 < 03
ADCC (cups/d, mean ± SD)	1.4 (1.2)	0.0 (0.0)	1.3 (0.5)	3.4 (1.1)	<0.001	1 < 2 < 03
ALCC (cup-years, mean ± SD)	48.3 (54.0)	0.0 (0.0)	42.5 (29.8)	126.6 (68.3)	<0.001	1 < 2 < 3

Notes: SD, standard deviation; ALAC, amount of lifetime alcohol consumption; ALS, amount of lifetime smoking; DLCC, duration of lifetime coffee consumption; ADCC, average amount of daily coffee consumption; ALCC, amount of lifetime coffee consumption.

Low = 2 cups of coffee or less per day; High = more than 2 cups of coffee per day.

^a^One-way analysis of variance for continuous variables and chi-square test for categorical variables.

^b^Bonferroni post hoc comparisons.

**Table 2:** Multiple linear regression analyses on the association between coffee consumption and log-transformed white matter hyperintensities volume/intracranial volume (logV_WMH_).

	All (n = 492)			Men (n = 204)			Women (n = 288)		
	β	SE	p	β	SE	p	β	SE	p
MODEL A^*^									
ALCC (cup-years)	0.094	0.001	0.030	0.099	0.001	0.129	0.112	0.002	0.040
MODEL B^*^									
ADCC (cups/d)	0.126	0.052	0.008	0.067	0.069	0.336	0.209	0.077	0.001
DLCC (years)	0.003	−0.003	0.958	0.080	0.005	0.259	−0.073	0.005	0.233

Notes: β, standardized regression coefficient; SE, standard error; BMI, body mass index; DM, diabetes mellitus; CVD, cardiovascular disease; ALAC, amount of lifetime alcohol consumption; ALS, amount of lifetime smoking; GDS, Geriatric Depression Scale; PSQI, Pittsburgh Sleep Quality Index; CIRS, Cumulative Illness Rating Scale; ALCC, amount of lifetime coffee consumption; ADCC, average amount of daily coffee consumption; DLCC, duration of lifetime coffee consumption.

^*^Adjusted for age, sex, years of education, BMI, hypertension, DM, CVD, ALAC, ALS, GDS, PSQI, and CIRS.

Compared to men, women showed smaller ICV (men = 1680.2 ± 128.8 cm^3^, women = 1472.4 ± 107.6 cm^3^, p < 0.001) and WM volume (men = 477.1 ± 128.6 cm^3^, women = 453.5 ± 185.3 cm^3^, p = 0.097). Compared to men, women started to consume coffee later (p = 0.001) and showed lower ALCC, ADCC and DLCC (p = 0.001). As summarized in Table 2, ALCC was associated with logV_WMH_ in women (adjusted R^2^ = 0.207, standardized β = 0.112, p = 0.040; MODEL A). V_WMH_ increased by 0.071 cm^3^ as the coffee consumption increased by one cup-year. Among the two determinants of ALCC, ADCC was associated with logV_WMH_ (adjusted R^2^ = 0.225, standardized β = 0.209, p = 0.001) but DLCC was not (adjusted R^2^ = 0.225, standardized β = −0.073, p = 0.233; MODEL B). V_WMH_ increased by 2.00 cm^3^ as the average daily coffee consumption increased by one cup. The women who consumed more than 2 cups of coffee per day on average showed greater logV_WMH_ than those who consumed 2 cups of coffee per day or less as well as those who never consumed coffee (Fig. 1). ALCC was not associated with logV_WMH_ in men (adjusted R^2^ = 0.224, standardized β = 0.099, p = 0.129; Table 2; MODEL A). V_WMH_ increased by 0.032 cm^3^ as the coffee consumption increased by once cup-year. The associations of logV_WMH_ with ADCC (adjusted R^2^ = 0.226, standardized β = 0.067, p = 0.336) and DLCC (adjusted R^2^ = 0.226, standardized β = 0.080, p = 0.259) were not significant (Table 2; MODEL B). The results were not changed when we analyzed the subjects who did not have a history of hypertension separately (106 men and 127 women).

## Discussion

This study found that higher ALCC was associated with higher logV_WMH_ in cognitively normal elderly individuals in both sexes. Although 3 to 4 cups of coffee per day (providing 300–400 mg/d of caffeine) are generally regarded as safe for most healthy adults^6^, the participants who consumed an average of more than 2 cups of coffee per day had greater logV_WMH_ in late life than those who consumed less.

To our knowledge, this study is the first to show a positive association between ALCC and late life V_WMH_ in cognitively normal elderly individuals. To date, there have been few studies on the effects of lifetime coffee consumption on WMH in humans, with conflicting results. Araújo *et al*. found no association between coffee consumption and V_WMH_ in middle-aged adults^41^. Ritchie *et al*. reported that coffee consumption was associated with lower V_WMH_ among women^42^. However, neither study considered the duration of coffee consumption or lifetime cumulative coffee consumption, and instead quantified coffee consumption through ADCC alone. Furthermore Ritchie *et al*. did not adjust for other potential confounding factors, such as hypertension, DM, and CVD^42^.

Coffee consumption may increase the risk of WMH via several mechanisms. First, caffeine, at a dose that is equivalent to 2 cups of coffee, reduces cerebral blood flow^7,9–12^ and abolishes the protective effect of ischemic preconditioning in humans^8^. Endogenous adenosine dilates cerebral vessels by activating adenosine A_2A_ and A_2B_ receptors of cerebrovascular smooth muscle^15,43^. At a normal dietary level, caffeine competitively antagonizes these adenosine receptors^15^, reduces adenosine-induced vasodilation up to 70%^44^. The majority of previous human studies have consistently found that chronic consumption of caffeinated coffee causes increased vascular resistance^15^, arterial stiffness^16^ and cerebral vasoconstriction^12^, which result in a decrease in cerebral blood flow due to blocking of the adenosine receptors. A study of middle-aged adults reported that increased aortic stiffness was independently associated with a greater V_WMH_^45^. Therefore, prolonged heavy consumption of caffeinated coffee may result in chronic cerebral hypoperfusion, which, in turn, may contribute to an increased V_WMH_ in late life. Moreover, variations in the expression and/or distribution of adenosine receptor subtypes within the cerebrovascular system and other components of the neurovascular unit, such as astrocytes, which regulate the tone of cerebral arterioles^46^, might influence V_WMH_, directly or indirectly. Second, the pressor response to caffeine could partly explain the association between coffee consumption and V_WMH_. High blood pressure is a major independent risk factor for increased V_WMH_^17–21^, and population-based MRI studies have shown a linear association between systolic and diastolic blood pressure levels and severity of V_WMH_^18,19^. In considering the long-term effects of caffeinated coffee consumption on normotensive adult populations, two meta-analyses of randomized controlled trials have shown increased blood pressure: 2.4 mm Hg in systolic pressure and 1.2 mm Hg in diastolic pressure^14^ and 2.0 mm Hg in systolic pressure and 0.7 mm Hg in diastolic pressure^13^. In hypertensive individuals, those who habitually drinking ≥3 cups of coffee per day showed higher 24-h systolic and (beta: 3.25 mm Hg) diastolic pressure (beta: 2.24 mm Hg) than non-coffee drinkers^47^. Paraxanthine, a major metabolite of caffeine, also increases the blood pressure in humans^48^. A previous observational study^49^ and a randomized controlled trial^50^ both reported that improved blood pressure control delayed the progression of WMH in elderly participants. It is therefore plausible that the effect of long-term coffee consumption on blood pressure may have contributed to the observed V_WMH_ increases, at least in part, during chronic daily lifetime exposure. However, the association of coffee drinking with the risk of hypertension was conflicting even between the previous meta-analyses; an inverted J-shape increase in one study^51^ while a dose-dependent decrease in other studies^52,53^. Furthermore, a recent study reported that several caffeine metabolites, such as methyluric acid and methylxanthine, reduced the odds for hypertension in adult individuals^54^. These ingredients may counterbalance caffeine’s pressor effect above a certain level of consumption^13,54^. Thus, further experimental studies are warranted to elucidate the mechanisms that underlie the effect of coffee consumption on the risk of WMH. Although the participants who regularly consume coffee may develop a degree of tolerance to the pressor effect of coffee, several experimental trials have confirmed that the tolerance may be partial. Caffeine can still to increase systolic and diastolic blood pressure even following regular consumption^55^. Furthermore, the pressor response to caffeine is regained after a relatively short period of abstinence^56^. Lastly, large individual variations in the sensitivity to the effects of coffee and the genetic polymorphisms associated with the enzymatic breakdown of caffeine or adenosine receptor function^57^ may also partly mediate the effect of coffee consumption on the V_WMH_ increase. Although sleep apnea may also increase the risk of cerebral WMH^58^, neither the PSQI score nor the STOP questionnaire score was different by coffee intake in the current study. Therefore, the association between coffee and V_WMH_ in our sample may not be confounded by comorbid sleep apnea.

When the both sexes were analyzed separately, lifetime consumption of coffee was associated with cerebral WMH in late life, in women but not in men, although the total brain and WM volumes of women are smaller than those of men. This difference may be, at least in part, attributable to the differences in hormones and/or the sensitivity to caffeine between the sexes. Sex differences in response to caffeine emerge after pubertal development, and these responses differ across the menstrual cycle in postpubertal women^59^. Women show more toxic reactions to caffeine than do men, suggesting that men and women metabolize caffeine differently^60^. In women, the increases in blood pressure after caffeine administration was greater when estradiol levels were higher, indicating that the sex differences in the caffeine response between men and women may be mediated by differences in sex hormone levels^24^. Estradiol decreases the rate of caffeine clearance in old women by inhibiting cytochrome P450 1A2 (CYP1A2) activity^23^.

Although higher cumulative coffee consumption may have a detrimental effect on late life cerebral V_WMH_ as described above, previous cross-sectional and longitudinal studies have reported that regular coffee consumption is associated with lower risk of stroke, DM, and CVD, better cognitive performance and a reduced risk of late life cognitive impairment/decline and dementia^1–5^, although the association was not found in all cognitive domains investigated and there was a lack of distinct dose-response associations. Therefore, given the overall benefits of coffee consumption on brain health, it is recommended to have an adequate amount of coffee depending on age, sex, and health status.

This study has some limitations. First, we could not account for the other sources of caffeine, such as tea, soft drinks, energy drinks, and chocolate products. We did not examine the type and preparation method of coffee (e.g. boiled, filtered, etc.), and did not differentiate between decaffeinated and caffeinated coffee, which directly influence the amount of consumed caffeine. However, coffee has been identified as a major dietary source of caffeine^6^, and decaffeinated coffee is not commonly consumed in Korea^61^. Second, because the patterns and amounts of coffee consumption were evaluated using retrospective self-reports and were subject to measurement error, we cannot rule out the possibility of misclassification bias, which could lead to the over- or underestimation of the real amount of coffee consumed. Nevertheless, results from validation studies suggest that self-reported habitual coffee consumption can be assessed with high reproducibility and validity^62^. Third, although habitual patterns of drinking coffee remain relatively stable over time^63^, some hypertensive patients with uncontrolled blood pressure and women who are pregnant or breastfeeding are more likely to refrain from consuming coffee^3,64^. However, we did not obtain information regarding the changes in participants’ coffee-drinking habits in the current study. Finally, causal inferences from our results is limited due to relatively small sample size and retrospective cohort design of this study.

In conclusions, we found that an increased lifetime cumulative coffee consumption was associated with an increased V_WMH_ in late life. People who consumed an average of more than 2 cups of coffee per day had greater V_WMH_ than those who consume less. The coffee-associated increase in V_WMH_ was larger in women than in men. Given that coffee is consumed worldwide and WMH are common in community-dwelling old people^17,18^, we should be concerned with the potential adverse effects of lifetime coffee consumption on brain health in late life.

## Supplementary information

Supplementary Information

## Data Availability

The datasets generated during and/or analyzed during the current study are available from the corresponding author on reasonable request. All methods were performed in accordance with the relevant guidelines and regulations.
