# Supplementary material for: Association between lifetime coffee consumption and late life cerebral white matter hyperintensities in cognitively normal elderly individuals
Source: Sci Rep. 2020 Jan 16;10:421. doi: 10.1038/s41598-019-57381-z (PMC6965088; doi:10.1038/s41598-019-57381-z)

**Supplementary Information**

Article in *Scientific Reports*

**Association between lifetime coffee consumption and late life cerebral white matter hyperintensities in cognitively normal elderly individuals**

Jeongbin Park^1^, Ji Won Han^2^, Ju Ri Lee^2^, Seonjeong Byun^2^, Seung Wan Suh^2^, Jae Hyoung Kim^3,4^, and Ki Woong Kim^1,2,5,*^

^1^Department of Brain and Cognitive Science, Seoul National University College of Natural Sciences, Seoul, Korea

^2^Department of Neuropsychiatry, Seoul National University Bundang Hospital, Seongnam, Korea

^3^Department of Radiology, Seoul National University Bundang Hospital, Seongnam, Korea

^4^Department of Radiology, Seoul National University College of Medicine, Seoul, Korea

^5^Department of Psychiatry, Seoul National University College of Medicine, Seoul, Korea

*Corresponding author: [kwkimmd@snu.ac.kr](mailto:kwkimmd@snu.ac.kr)

**Methods**

**Coffee consumption questionnaire**

| 1. **“How many cups of coffee do you drink per day over the past year?”**   (Average amount of daily coffee consumption over the past year [current ADCC, cups/day]) |
| --- |
| 1. **“How many cups of coffee do you drink per day during your lifetime?”**   (Average amount of daily coffee consumption during lifetime [ADCC, cups/day]) |
| 1. **“At what age did you start drinking coffee?”**   (Age at the start of coffee drinking [years]) |
| 1. **“At what age did you end drinking coffee?”**   (Age at the end of coffee drinking [years]) |


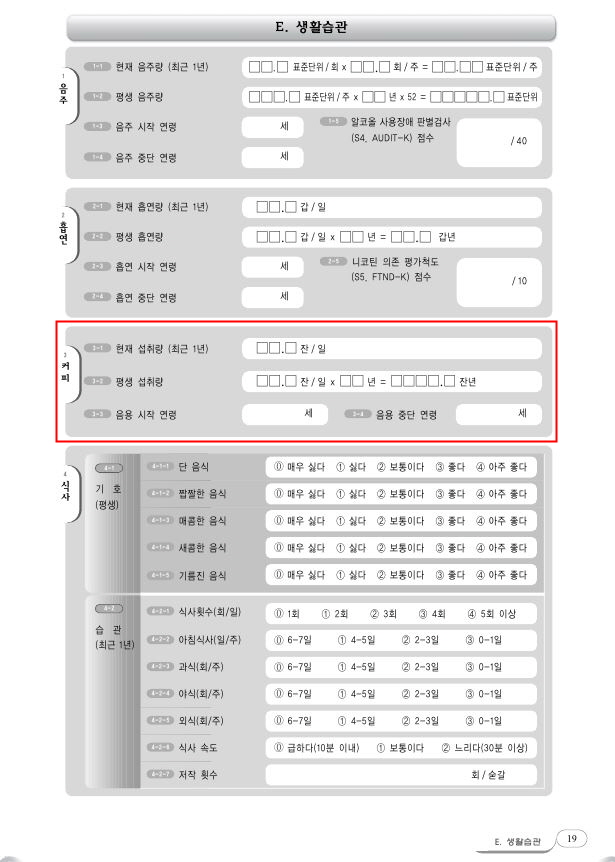

Supplement: Supplementary file 1 — Supplementary Information [file 41598_2019_57381_MOESM1_ESM.docx]
